# Transtheoretical Model Is Better Predictor of Physiological Stress than Perceived Stress Scale and Work Ability Index among Office Workers

**DOI:** 10.3390/ijerph17124410

**Published:** 2020-06-19

**Authors:** Maria Marin-Farrona, Manuel Leon-Jimenez, Jorge Garcia-Unanue, Leonor Gallardo, Carmen Crespo-Ruiz, Beatriz Crespo-Ruiz

**Affiliations:** 1 IGOID Research Group, Department of Physical Activity and Sport Sciences, University of Castilla-La Mancha, 45071 Toledo, Spain; mmarinfa@gmail.com (M.M.-F.); manuel.leonjimenez@yahoo.es (M.L.-J.); leonor.gallardo@uclm.es (L.G.); beatriz.crespo@uclm.es (B.C.-R.); 2 Freedom and Flow Company, 28050 Madrid, Spain; carmen.crespo@freedomandflowcompany.com

**Keywords:** Transtheoretical Model, heart rate variability, physical activity, stress, office workers, Work Ability Index, Perceived Stress Scale

## Abstract

Physical activity impacts positively on stress and anxiety. Working conditions affect the quality of life by increasing stress levels, which can affect job performance and work absence. The Perceived Stress Scale (PSS), Work Ability Index (WAI), Transtheoretical Model (TTM), as well as heart rate variability (HRV) have been applied to monitor the state of workers in their job. The aim of this study was to analyze PSS, WAI, and TTM classifications, and to find out how are they linked to physiological stress (HRV). One hundred and thirteen office workers responded to the three questionnaires and their HRV was monitored for at least two full days. Groups were set up according to TTM (Stage 1, Stage 2, Stage 3–4, Stage 5), WAI (weak WAI, medium WAI, good WAI), and PSS (low PSS, medium PSS, high PSS). Results obtained from the test were related to stress values measured by HRV with a Bodyguard2 device. The Stage 5 group from TTM had better HVR and stress levels than the other groups for both women and men (*p* < 0.05). Participants in the good WAI group and low PSS group had better results than weak WAI and high PSS, but the differences with respect to medium WAI and medium PSS were less clear. Finally, TTM seemed to be the best tool to discriminate physiological stress in office workers with regard to other questionnaires.

## 1. Introduction

Low investments in health and safety promotion carried out by companies can have a huge impact on workers. On the one hand, there is an evident relationship between wellbeing and general positive health indicators, such as job satisfaction, the quality of life, morbidity, and productivity [1]. On the other hand, the World Health Organization (WHO) define health as “a state of complete physical, mental and social wellbeing and not merely the absence of disease or infirmity” [2]. In accordance with this definition, companies promote initiatives that include a variety of physical wellbeing dimensions worthy of assessment. Among others, these include professional satisfaction, fatigue, burnout, engagement, emotional stress, and several factors influencing the quality of life, such as physical activity, nutrition, or social health [3]. One of the reasons is that they are usually connected. For example, physical activity and exercise have been demonstrated to promote positive changes in one’s mental health and ability to cope with stressful encounters [4].

Stress is considered the silent disease of the 21st century [5]. Due to the increasing identification of mental ill-health in the workplace, for the first time, the World Health Organization has classified “burnout” as an “occupational phenomenon” in the 11th revision of the International Classification of Diseases [6]. Mental illness such as burnout, stress, post-traumatic stress disorder, anxiety, and depression are not only manifested in relationships, families, or academic responsibilities, but also in the healthcare workplace [7].

Stress does not have one particular cause. The human stress response has evolved to maintain homeostasis under conditions of real or perceived stress. Our organism will respond in the way it deems most appropriate to face the situation as soon as possible [8]. Potential threats generate physiological responses like high heart rate, changes in insulin levels, sweating, high blood pressure, blood vessel dilation, pupil dilation, and cognition [9]. Accordingly, cognitive and behavioral reactions such as the inability to make decisions and concentrate, frequent forgetfulness, hypersensitivity to criticism, mental block, impulsive behavior, depression, fatigue, irritability, and low self-esteem are also manifested. These can directly impact day-to-day living, and more specifically, taking into account the characteristics of this study, the hours of absenteeism and productivity [10]. To this effect, there are numerous challenges that companies must face when it comes to improving wellbeing and health. The rise of pathologies such as anxiety, stress, and depression, has led to financial losses up to €136 million in companies due to the percentage of workers in the European Union who suffer daily stress reaching more than 75% [11].

Moreover, the great transformations in the global labor markets, labor relations, economic and market globalization, the financial crisis, technological changes, and demographic and social changes in different regions of the world are affecting the organizational forms of companies and their workers, including new working methods such a teleworking [12]. As a result, human resource departments in companies are carrying out initiatives to identify factors that may affect the wellbeing of workers. Thus, they have tried to quantify stress objectively to understand the influence of this variable on the health status of workers [13]. For example, in recent years the search for instruments to objectively quantify stress has been focused on heart rate variability (HRV), which is the variation in the RR-intervals (or between heartbeat and heartbeat); it reflects many factors that can regulate the heart rate, which is modulated by the nervous system (sympathetic and parasympathetic system) [14]. The sympathetic activity tends to increase heart rate and decrease HRV because of the release of epinephrine and norepinephrine, while parasympathetic activity reduces heart rate and increases HRV by the release of acetylcholine from the vagus nerve. Consequently, the measurement of parasympathetic tone may serve as an index of stress and stress vulnerability due to the heart rate and rhythm being largely under the control of the autonomic nervous system. Finally, HRV can be used as a useful instrument to measure the sympathetic and parasympathetic function of the autonomic nervous system [5].

However, as a result of the high complexity of quantifying stress objectively, due to measuring implications and the requirement for the workers, different questionnaires have been applied, showing difficulties due to their multidimensional subjective stress factors. For example, the Work Ability Index (WAI) is a questionnaire based on the capacity of employees to carry out their work and their projection over the next two years, taking into account the demands of the job, the state of health, and resources of the workers [15,16]. It was developed by the Finnish Institute of Occupational Health and has been translated into 25 different languages [17]. The WAI is applied in health promotion programs to maintain and promote worker participation and improve work performance [18]. Other researchers concluded that lower rates of work capacity are linked to longer-term sick leave [19]. On the one hand, it was shown that regular exercise, educational level, and sleep quality are associated with better scores in the WAI [20,21]. Furthermore, questionnaires like the Perceived Stress Scale (PSS) have also been used to find the subjective stress levels of the workers, but data obtained refer only to the last month and stress is a state which changes continually [22,23]. Perceived stress is a psychological concept that has received much attention over the last two decades. Some researchers have shown that the perception of work-related stress, measured by the PSS significantly impacted the quality of life of workers [24].

Finally, the Transtheoretical Model (TTM) created by Prochaska and DiClemente defines “The stages of change” [25]. It is a questionnaire based on the argument that behavioral changes follow a series of standardized stages. The TTM has been used in several behavioral change studies related with physical exercise and as a strategy to control multifactorial morbidities, including chronic diseases, eating habits and/or engaging in physical activity, and others [26,27]. We consider it interesting to include TTM questionnaire because exercise and physical activity are associated with less subjective stress. It has been demonstrated in randomized clinical trials that have determined exercise as an effective method for improving perceived stress, stress symptoms, and quality of life [28].

For these reasons, the aim of this study was to analyze each of the previously described questionnaires that measure psychological stress (PSS), work ability (WAI) and the behavioral changes, which is influenced by physical activity (TTM), and to find out how are they linked to physiological stress, in order to facilitate the measurement of this variable in future companies and thus find out the state of mental health of their workers by applying a questionnaire.

The study hypothesizes that participants who intend to practice physical activity or who have already been practicing physical activity in the last 6 months, as measured by the TTM, will show fewer physiological stress values due to the benefits associated with physical activity. Similarly, those who belong to the high PSS group and weak WAI group will show higher levels of physiological stress.

## 2. Materials and Methods

### 2.1. Sample

The study sample was composed of 113 office workers (*n* = 113); of this total, 66 were women and 47 were men. All of them were invited to participate voluntarily and were recruited by different office departments of three companies based in Spain. The participants had an age of 38.85 ± 5.80 years for women and 38.57 ± 5.59 years for men. In regards to height, 1.72 ± 0.06 m for women and 1.73 ± 0.06 m for men. Concerning weight, 71.17 ± 6.68 kg for women, and 74.00 ± 5.79 kg for men. Finally, Body Mass Index (BMI) values were 23.83 ± 2.22 for women, and 24.64 ± 2.00 for men.

The participants did not have any cardiac illness, high blood pressure, diabetes or any other chronic illnesses, following the method of previous studies [29]. The subjects were informed of the use of the data for this study and accepted the continued monitoring of the HRV for at least 48 h of registered data through signed consent. Inclusion criteria were based on a previous study and included a minimum of 4.5 h beat-to-beat R-R interval recording during sleep after a workday [30]. Another inclusion criterion was the availability of RR-interval data, including at least one workday (≥4 h of work) and the analyzed data consisted of successfully recorded when measurement error was <15%, and there was <30 recording breaks in each of the 24 h of recording. Participants who had consumed alcohol on the monitoring days were excluded. Therefore, 12 participants had to be excluded from a total of 125 participants. The study complies with the ethics committee (Health Sciences Research Committee of European University, CIPI/045/16), based on the Helsinki declaration.

### 2.2. Transtheoretical Model (TTM)

The complete sample was classified into four groups based on the TTM questionnaire. The TTM emerges across a variety of theories and models of behavior change. TTM uses the stages as a temporal dimension to determinate the acquisition and maintenance of behavior in order to adopt and maintain changes in their lifestyle. This instrument has been created by Prochaska and DiClemente [31], and it has also been validated in other intervention studies which have focused on smoking cessation, stress management, diet, and exercise [25]. For example, interventions related to the acquirement and preservation of exercise behavior to adopt exercise as an integral part of a healthier lifestyle [32].

This instrument consists of five stages of change, which depend on intentions and behavior changes. In Stage 1 (pre-contemplation stage), individuals do not want to make a behavior change in the near future. In Stage 2 (contemplation stage), individuals think about making a health behavior modification but have not yet decided to change. In Stage 3 (preparation stage), individuals want and are ready to make the behavior change. When the behavior stage is initiated and this change has occurred for less than 6 months, it is determined as the Stage 4 (action stage). Stage 5 (maintenance stage) is reached when behavior change has been long-lasting. In this study, we grouped the stages 3 and 4, due to the limited influence of physical activity on HRV in short periods (P–A) [33]. Each participant answered the short version of TTM questionnaire, which included five clauses (Stage 1; I am not currently physically active or active, and I am not planning to change; Stage 2; I am thinking of doing physical activity in the next few days; Stage 3; I am active, but not regularly; Stage 4; I am currently active, but I started physical activity less than 6 months ago; Stage 5; I am currently active or active and have been practicing physical activity for 6 months), and were assigned to a one of the four stages.

### 2.3. Work Ability Index (WAI)

The sample was classified into three groups according to WAI, which is a questionnaire used in occupational health and research to assess work ability of workers during health examinations and workplace surveys [34]. The index is determined based on the answers to a series of questions, which take into consideration the demands of work, the worker’s health status, and resources. The questionnaire has seven dimensions, including current working capacity compared to workers’ best lifetime period of work; work capacity, according to the physical and mental nature of work; a number of diseases diagnosed by a doctor; personal thoughts about work disability due to illness; sick leave for the last 12 months, a personal prediction of work capacity for the next two years; and an estimation of mental problems related to the disease. The WAI consists of 10 questions arranged in seven items ranging from 7–49 with higher values indicating better work ability. The results are classified into four groups, which are weak work ability (weak WAI; 7–27), medium work ability (medium WAI; 28–36), good work ability (good WAI; 37–43), and excellent work ability (excellent WAI; 44–49) [16]. In this study, good WAI and excellent were grouped in the good WAI due to the low number of participants included in them. The validity and reliability of the WAI were assessed in correlation analyses. More recently, the validity of the WAI has been shown in different research [34].

### 2.4. Perceived Stress Scale (PSS)

The PSS is the most widely used psychological instrument for measuring the perception of stress. The tool, while originally developed by Cohen, in 1983 [35], remains a popular choice for helping people to understand how different situations affect their feelings and their perceived stress. The validity of the PSS has been shown in recent research [36]. The questions in this scale ask about feelings and thoughts during the last few months. Items were designed to tap how unpredictable, uncontrollable, and overloaded respondents find their lives. The participants had to indicate how often they experienced a certain way.

Some researchers have shown correlations with PSS and stress measures, self-reported health and health-service measures, smoking status, and help-seeking behavior [35]. The PSS is composed of 14 items rated on a Likert scale (from 0 to 4). Total score from the sum of questions may vary from 0 to 40 and the perceived stress is higher as scores increase. The PSS is classified into three groups, which are low perceived stress (low PSS; 0−13), medium perceived stress (medium PSS; 14−26), and high perceived stress (high PSS; 27−40) [37].

### 2.5. Heart Rate Variability (HRV)

In this study, all the participants recorded their HRV in order to compare physiological stress with the results obtained by WAI, PSS, and TTM. HRV is the fluctuation of the duration of heartbeat intervals, it represents the ability of the heart to respond to a different stimulus, and it can be used to measure the response of the autonomic nervous system in different situations [5]. Thus, using the Firstbeat Bodyguard 2 device (Firstbeat Technologies Ltd., Jyväskylä, Finland), a non-invasive device, ambulatory beat-to-beat RR-intervals recorded were used to determinate the root mean square of the successive differences (RMSSD) and a load of stress (%Stress) and recovery (%Recovery) [38].

In order to obtain the data, the heart rate, which is controlled by the parasympathetic nervous system and sympathetic nervous system, was measured in beats per minute. Furthermore, the analysis of RMSSD was measured in milliseconds for 48 h. Recent research has shown how acute stress correlates with a decrease in HRV during sleep and during the day, finding a strong relationship between the decrease in HRV and work stress [39]. Thus, during the sleeping time collection, the first half-hour was excluded, and the analyses of the following 4 h were done during the slow-wave sleep, which usually takes place during this time. From these parameters, RMSSD was used as an indicator of vagal cardiac control. With this information, we could understand how the parasympathetic nervous system affects the organism; the greater the RMSSD, the greater the parasympathetic activity [39,40].

**Figure 1:**
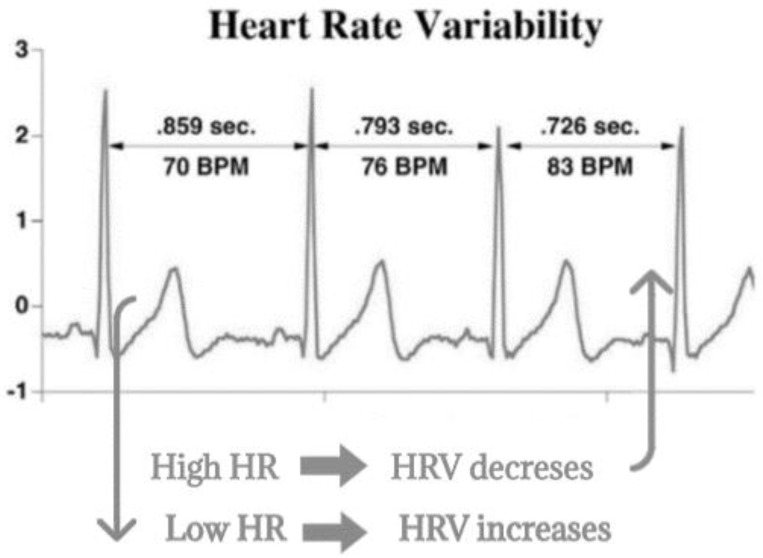
Heart rate variability interpretation. Abbreviations: BPM, Beats per Minute; HR, Heart Rate; HRV, Heart Rate Variability.

When the organism feels stressed, our sympathetic nervous system increases the heart rate (HR) and consequently, the time between successive RR-intervals gets shorter, so that HRV decreases. Conversely, when the organism is relaxed HR is decreased by our parasympathetic nervous system and the time between successive RR-intervals gets longer thus HRV increases. This fluctuation in time between RR-intervals is called the HRV (Figure 1).

Finally, we obtained: a total stress time in 24 h; stress percentage (%Stress), which is the percentage of reaction to stress in an average of 24 h; recovery time in 24 h, which includes the time in which the body is recovering; and the recovery percentage in 24 h (%Recovery). All these variables are assessed in minutes per 24 h. The recollection period of the data was between two and three continued workdays. Finally, we analyzed only 48 h of full data. This information was downloaded to the Firstbeat SPORTS software (version 4.7.2.1, Firstbeat Technologies Ltd., Jyväskylä, Finland), which offers the possibility of exporting the variables to a database. The validity of the Bodyguard 2 device has been assessed in other research, which demonstrated it as an accurate device for monitoring HRV [41]. Finally, all the variables were related between them. %Stress and %Recovery were obtained from the RMSSD variable. The HRV variables needed for state detection were time domain and frequency domain HRV variables, such as RMSSD [38].

### 2.6. Other Data

At the beginning, participants filled out a brief questionnaire about their hours of physical activity per week. During the measurement, the participant completed a diary, which included information about workdays, working hours, days off, and sleep periods.

### 2.7. Statistical Analysis

Data encoding and data processing were carried out using the SPSS 25.0 statistical package (SPSS Inc., Chicago, IL, USA). The normality of the variables has been analyzed with the Kolmogorov–Smirnov statistic. After a descriptive analysis (means and standard deviations), a comparison test was performed by the analysis of covariance (ANCOVA). Age and BMI were used as covariables because of their influence in the HRV parameters and the inter-individual variability [42]. TTM, WAI, and PSS groups were treated as factors, and RMSSD, %Recovery, and %Stress as dependent variables. A Bonferroni post-hoc test was used for pairwise comparisons. In addition, the confidence interval and effect size (ES; Cohen’s d) have been included. The ES was evaluated as follows: 0–0.2 = trivial; 0.2−0.5 = small; 0.5−0.8 = moderate; and >0.8 high. Finally, several regression estimations were performed to analyze the influence of TTM, WAI, and PSS classifications on the different physiological stress variables. All models were also controlled by sex, age, and BMI. The regression did not present normality or heteroscedasticity problems. Moreover, the Variance Inflation Factor (VIF) did not report any multicollinearity problems. The statistical significance criterion was established at *p* < 0.05.

## 3. Results

Descriptive and anthropometric data for women and men are shown (Table 1). The results referring to weight and BMI show significant differences between women and men (*p* < 0.005; *p* = 0.001). On the other hand, no significant differences were observed between the hours of physical activity, age, and height between men and women.

**Figure 2:**
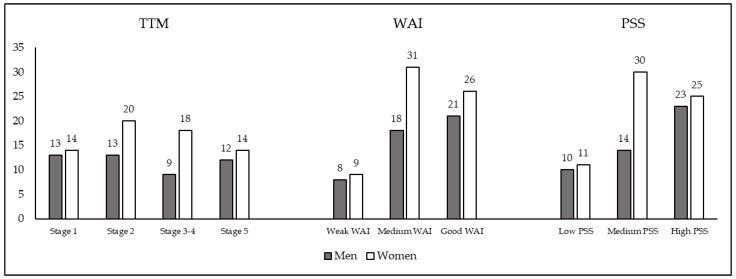
The number of participants in each classification. Abbreviations: TTM, Transtheoretical Model; WAI, Work Ability Index; PSS, Perceived Stress Scale.

The sample of men and women were classified into four groups according to TTM, in three groups according to WAI, and three groups according to PSS. Figure 2 represents the distribution of the sample in each classification. It is important to note that the whole sample (46 men and 66 women) is included in each classification.

Table 2 shows the differences in physiological stress between the four TTM stages. The main effect reported a significant difference for RMSSD, %Stress, and %Recovery (*p* < 0.001 in men and women). Post-hoc comparisons showed that TTM Stage 5 reported higher values of RMSSD and %Recovery and lower values of %Stress that the other three TTM stages, both in men and women (*p* < 0.05; ES from 0.486 to 1.58). No differences were found between Stage 1, Stage 2, and Stages 3 and Stage 4 groups (*p* > 0.05).

Table 3 shows the differences in physiological stress between the three groups of the WAI classification. The main effect showed a significant difference for RMSSD (*p* < 0.001 in men and women), %Stress (*p* = 0.039 in men and *p* = 0.043 in women), and %Recovery in men (*p* = 0.006). No main effects were found in women (*p* > 0.05). Regarding post-hoc comparisons, good WAI reported higher values of RMSSD than the medium WAI and weak WAI groups in men and women (*p* < 0.01; ES from 0.87 to 1.15). In %Stress, differences between good WAI group and weak WAI group were found in men and women (*p* < 0.05; ES: from 1.35 to 1.36). Men showed lower values in %Recovery in good WAI group than medium and low WAI groups (*p* < 0.01; ES: from 1.08 to 1.65).

Table 4 shows the differences in physiological stress between the three groups of the PSS classification. The main effect reported a significant difference for RMSSD (*p* = 0.001 in men and *p* < 0.001 in women), %Stress (*p* < 0.001 in men and women), and %Recovery (*p* < 0.001 in men and women). After post-hoc comparisons, high PSS reported higher values of RMSSD than the low PSS group in men and women (*p* < 0.001; ES from 0.95 to 1.21). In %Stress, differences between high PSS group and low PSS group were found in men and women (*p* < 0.001; ES: from 2.98 to 3.75). Finally, high PSS reported higher values of %Recovery than the low PSS group in men and women (*p* < 0.01; ES from 3.38 to 3.75).

Finally, Table 5 report the results of the regression estimations, analyzing the influence of TTM, WAI, and PSS classifications on the different physiological stress variables. TTM classification is the only one with significant influence in all models (*p* < 0.01). Stage 5 TTM presents a positive and significant relationship in RMSSD and %Recovery and a negative relationship with %Stress. On average, regardless of the other variables, participants in Stage 5 of TTM would have 15.88 RMSSD, 7.89% less stress and 6.91% more recovery. PSS also had a significant influence in %Stress, with a positive relationship, and %Recovery, with a negative relationship (*p* < 0.05). However, WAI did not show any significant influence in %Stress, %Recovery, or RMSSD.

## 4. Discussion

This study aimed to compare physiological stress measured by HRV between the groups classified through the questionnaires state of change as a result of TTM, work ability measured by WAI, and psychological stress from PSS; thus, to understand which of these three questionnaires is able to better discriminate the level of physiological stress. The study hypothesized that participants with less physiological stress could be represented through Stage 5 of TTM, high values from WAI, and lower parameters from PSS.

The results show the TTM as a consistent and discriminatory questionnaire that represents physiological stress with regard to the other questionnaires. Accordingly, the Stage 5 group has better values than the other groups (Stages 1,2,3–4) in the variables %Stress, %Recovery, and RMSSD. Moreover, the relation between both WAI and physiological stress, and PSS and physiological stress results were analyzed. In those questionnaires, the good WAI group and low PSS group had better results in the variables analyzed (%Stress, %Recovery, and RMSSD), showing lower levels of physiological stress, but results are not as clear with respect to the TTM, because less significant differences between high PSS and medium PSS were found, as well as fewer differences were found in good WAI respect to medium WAI.

Principal results suggest that men and women who practice physical activity regularly belonging to the Stage 5 group, have a better HRV than those participants who do not practice physical activity regularly. Therefore, TTM seems to discriminate HRV and percentages of stress and recovery explicitly when positioning in Stage 5. Furthermore, people with good WAI have better HRV. These results coincide with previous research which has shown the relationship between physical activity, HRV, and work capacity [43]. Conversely, other studies have demonstrated that physical activity programs in the workplace or home, promoted by companies, improve work capacity in employees. Apart from that, monitoring work capacity for the long-term can predict absenteeism [44,45]. However, data recording can be influenced by subjectivity and the period in which stress is measured [46], because work ability is a changing state. When somebody begins a job that involves new challenges, the perception of work capacity will be low, since these are new tasks demanded. However, when somebody feels competent to perform a job, the work ability perception is high, but they still face new stimuli and that can increase stress levels. For this reason, the perception of work capacity can be linked to physiological stress, both when they do not feel competent to carry out the tasks, or when even knowing that they have the skills to face some tasks, they have to manage their efforts and time to carry them out. Finally, it is positive physiological stress of adaptation to work that allows improvement [47].

This fact could explain the few differences between high and medium WAI. Future research should analyze working capacity over time, and not just interpret one score, due to previous reports showing a significant association between the changes in WAI score and changes in work environments. Therefore, observing a decrease in the WAI score measured over time may lead to greater accuracy in the ability to predict the absence of disease [47].

Regarding PSS, the low PSS group had high HRV. These results make sense due to the relation between physiological stress and psychological stress. Conversely, people with high-stress levels show a reduction in their HRV parameters due to the inhibition of the parasympathetic nervous system [48]. Additionally, attending to the effect size on HRV, practicing physical activity regularly has a greater influence on HRV, and this will be reflected in the perception of the worker with an excellent work capacity measured by the WAI. Interestingly, an initially higher level of physical activity associated with a larger decline in subjective stress has been described [49]. This suggests that physical activity has beneficial effects on the individual’s ability to decrease the level of subjective stress. To clarify, physically active individuals who at the same time experience stress are more inclined to lower the level of subjective stress compared with those who are psychologically stressed and inactive. Likewise, a greater amount of sedentary behavior is associated with higher levels of perceived stress among older adults has been demonstrated [50].

TTM is one of the most commonly used methods in behavioral change modelling. It has been applied in different research with the purpose of understanding the influence of physical activity habits. For example, attending to the relationship observed between physical activity and HRV, different stress response according to the TTM have been shown [51]. In particular, participants who have practiced physical activity regularly for 6 months can manage stress levels, showing better autoregulation skills than people who do not practice exercise. Along the same lines, TTM can prevent chronic diseases [52]. Other researchers investigated the influence of labor seniority, job category, work engagement, and burnout syndrome in HRV, finding a positive association that is also related to TTM [53].

These results suggest that using HRV at the workplace can indicate burnout syndrome, stress levels, or wellbeing in workers. As a consequence, promoting health programs in a corporation could have a better effect. Indeed, a 1-min paced deep-breathing HRV protocol has been applied in the workplace as part of a comprehensive preventive workers’ health assessment [54]. Other research concluded there is a negative effect of stress on obesity, absenteeism, and productivity [55]. Finally, those conclusions support the use of occupational health programs that include nutritional and physical activity initiatives in companies to prevent stress in office workers. The use of the TTM with workers could offer important information about their health situation. Thus, the company could promote activities such as stress management, physical activity, psychological therapy, or nutrition education, among others, to improve the health of workers.

All the benefits support the studies on physical activity programs focused on personal differences (states of change, smoking, work–life balance), which include Heart Rate monitoring, to improve stress management for workers. For example, a physical activity program consisting of HRV biofeedback-based training has shown benefits in reducing stress levels [56]. Other programs include activities focused on diaphragmatic breathing, relaxation, meditation, and emotional intelligence [56]. Closely linked with this, it has been demonstrated that levels of anxiety and depression can be predicted by technological devices [57]. However, it is important to ensure the workers do not perceive this monitoring negatively; although technology can help with data collection, sometimes people do not react positively to e-health intervention [58].

The possibility of people going backwards in the stages of change exists. In this sense, motivation plays a primary role and is considered a determining element in TTM [59,60]. Self-determination theory is a macro-theory of human motivation that has been applied to health-relevant change. This theory distinguishes between no auto-determined motivation and auto-determined motivation [61]. Recently, research has concluded that greater auto-determined motivation is positively related to a high commitment and adherence to sports practice, and therefore with the Stages 4 and 5 from TTM [62,63]. Furthermore, physical activity has a protective effect on stress [12]. Therefore, it is proposed as a tool to improve the health status of workers, as well as to improve their emotional state and increasing self-determined motivation.

There are several limitations to this project. First, the total number of the days measured only included working days. The introduction of more days of data collection, including weekends, would have provided more information, although it would greatly increase the complexity of the results. Second, although the position of the electrodes was controlled by the investigators and all the subjects were informed, and instructions were provided, there is an implicit bias to possible displacement of the device. Finally, the 24-h registration inclusion criterion made it very difficult to participate in the study. Therefore, the final sample has not been high, being one of the main limitations of this research.

## 5. Conclusions

The three analyzed questionnaires represent physiological stress. However, TTM is the best tool to discriminate physiological stress in office workers regarding other questionnaires (WAI and PSS). The intention of practicing physical activity was positively reflected in the Stage 5, as it influences a decrease in physiological stress levels. In addition, it represents an advance in the application of different questionnaires in work environments due to its easy applicability. Using just one questionnaire, we can obtain relevant information related to physiological stress on workers. In this sense, we can offer relevant information on the health status of workers, as well as promote healthy initiatives in companies. Future research could analyze the effects of a training program based on HR biofeedback specifically for office workers, to understand whether this type of training method could improve the health and stress levels of the employees.

## Figures and Tables

**Table 1:** Mean ± SD according to sex; age; height (m); weight (kg); BMI; PA hours; PA status.

Variables	Women (*n* = 66)	Men (*n* = 47)	*p*-Value
Age	38.85 ± 5.80	38.57 ± 5.59	0.673
Height (m)	1.72 ± 0.06	1.73 ± 0.06	0.437
Weight (kg)	71.17 ± 6.68	74.00 ± 5.79	>0.000
BMI	23.83 ± 2.22	24.64 ± 2.00	0.001
Hours of physical activity	1.39 ± 2.09	1.53 ± 2.34	0.545

Abbreviations: SD, Standard Deviation; BMI, Body Mass Index; PA, Physical Activity.

**Table 2:** Differences in RMSSD, %Stress and %Recovery in the different TTM groups.

		Stage 1	Stage 2	Stage 3–4	Stage 5
Men	RMSSD (ms)	64.92 ± 11.42	64.00 ± 13.80	62.44 ± 11.79	81.40 ± 12.27 ^a,b,c^
	%Stress	69.79 ± 2.76	70.62 ± 3.84	69.11 ± 6.14	59.21 ± 3.04 ^a,b,c^
	%Recovery	23.92 ± 2.85	21.95 ± 2.86	24.52 ± 4.59	35.58 ± 3.02 ^a,b,c^
Women	RMSSD (ms)	60.01 ± 9.32	60.98 ± 11.40	65.38 ± 11.33	71.55 ± 13.95 ^a,b,c^
	%Stress	72.06 ± 3.08	71.89 ± 2.76	68.56 ± 5.34 ^b^	60.65 ± 2.99 ^a,b,c^
	%Recovery	23.32 ± 5.70	22.35 ± 2.87	26.30 ± 5.18 ^b^	33.69 ± 3.83 ^a,b,c^

Abbreviations: RMSSD, the root mean square of the successive differences; TTM, Transtheoretical Model; Stage 1, pre-contemplation stage group; Stage 2, contemplation stage group; Stage 3–4, preparation and action group; Stage 5, maintenance stage group. ^a^ differences with respect to group Stage 1 (*p* < 0.05); ^b^ differences with respect to Stage 2 (*p* < 0.05); ^c^ differences with respect to Stage 3–4 (*p* < 0.05).

**Table 3:** Differences in RMSSD, %Stress and %Recovery in the different WAI groups.

		Weak WAI	Medium WAI	Good WAI
Men	RMSSD (ms)	63.63 ± 11.62	61.83 ± 12.78	75.85 ± 13.29 ^a,b^
	%Stress	71.29 ± 2.49	68.57 ± 3.32	64.44 ± 7.59 ^a^
	%Recovery	22.38 ± 2.25	24.04 ± 4.13	30.11 ± 7.08 ^a,b^
Women	RMSSD (ms)	57.30 ± 10.17	60.87 ± 9.47	70.62 ± 12.94 ^a,b^
	%Stress	72.40 ± 2.34	69.45 ± 4.93	66.37 ± 6.58 ^a^
	%Recovery	23.26 ± 3.09	24.90 ± 5.89	28.36 ± 6.50

Abbreviations: RMSSD, the root mean square of the successive differences; WAI, Work Ability Index; ^a^ differences with respect to weak WAI (*p* < 0.05); ^b^ differences with respect to Medium WAI (*p* < 0.05).

**Table 4:** Differences in RMSSD, %Stress and %Recovery in the different PSS groups.

		Low PSS	Medium PSS	High PSS
Men	RMSSD (ms)	77.10 ± 7.70	64.88 ± 16.47	66.76 ± 14.01 ^a^
	%Stress	58.80 ± 3.08	68.64 ± 4.74 ^a^	69.94 ± 4.38 ^a^
	%Recovery	35.85 ± 3.26	24.50 ± 5.47 ^a^	23.58 ± 3.26 ^a^
Women	RMSSD (ms)	76.00 ± 12.29	61.38 ± 11.38 ^a^	62.43 ± 10.06 ^a^
	%Stress	59,86 ± 2,27 ^b^	69.06 ± 4.86 ^a^	71.98 ± 3.33 ^a,b^
	%Recovery	34.63 ± 3.25	25.72 ± 4.92 ^a^	22.64 ± 4.61 ^a^

Abbreviations: RMSSD, the root mean square of the successive differences; PSS, Perceived Stress Scale; ^a^ differences with respect to low PSS (*p* < 0.05); ^b^ differences with respect to medium PSS (*p* < 0.05).

**Table 5:** Influence of TTM, WAI, and PSS classifications on physiological stress.

	RMSSD (ms)		%Stress		%Recovery	
Men (reference group)						
Women	−3.84	(2.02)	1.27	(0.77)	−0.26	(0.82)
Age	−1.06	(0.18) **	0.10	(0.07)	−0.03	(0.07)
BMI	−0.55	(0.46)	0.04	(0.18)	−0.09	(0.19)
Stage 1 TTM (reference group)						
Stage 2 TTM	3.77	(3.08)	0.57	(1.17)	−1.92	(1.25)
Stage 3–4 TTM	3.68	(3.51)	−1.53	(1.33)	0.96	(1.43)
Stage 5 TTM	15.88	(5.01) **	−7.89	(1.90) **	6.91	(2.04) **
Weak WAI (reference group)						
Medium WAI	0.71	(3.29)	−1.49	(1.25)	0.62	(1.34)
Good WAI	6.15	(3.67)	−0.43	(1.39)	0.68	(1.49)
Low PSS (reference group)						
Medium PSS	1.63	(4.27)	3.65	(1.62) *	−3.65	(1.74) *
High PSS	2.80	(4.60)	4.53	(1.75) *	−4.86	(1.87) *
Constant	116.07	(14.02) **	60.54	(5.32) **	31.89	(5.70) **
R^2^	0.50		0.64		0.62	
F	10.10 **		18.25 **		16.88 **	

Abbreviations: RMSSD, the root mean square of the successive differences; BMI, Body Mass Index; TTM, Transtheoretical Model; WAI, Work Ability Index; PSS, Perceived Stress Scale * *p* < 0.05; ** *p* < 0.01.
